# Posthypoxic behavioral impairment and mortality of *Drosophila melanogaster* are associated with high temperatures, enhanced predeath activity and oxidative stress

**DOI:** 10.1038/s12276-021-00565-3

**Published:** 2021-02-09

**Authors:** Pardes Habib, Jennifer Jung, Gina Maria Wilms, Alma Kokott-Vuong, Shahin Habib, Jörg B. Schulz, Aaron Voigt

**Affiliations:** 1 Department of Neurology, Medical Faculty, RWTH Aachen University, 52074 Aachen, Germany; 2 Institute of Biochemistry and Molecular Immunology, Medical Faculty, RWTH Aachen University, 52074 Aachen, Germany; 3 JARA-BRAIN Institute Molecular Neuroscience and Neuroimaging, Forschungszentrum Jülich GmbH and RWTH Aachen University, 52074 Aachen, Germany; 4 Medical Biochemistry, Department of Biochemistry, University of Leicester, Leicester, United Kingdom

**Keywords:** Stroke, Molecular neuroscience

## Abstract

Hypoxia is an underlying pathophysiological condition of a variety of devastating diseases, including acute ischemic stroke (AIS). We are faced with limited therapeutic options for AIS patients, and even after successful restoration of cerebral blood flow, the poststroke mortality is still high. More basic research is needed to explain mortality after reperfusion and to develop adjunct neuroprotective therapies. *Drosophila melanogaster (D.m.)* is a suitable model to analyze hypoxia; however, little is known about the impacts of hypoxia and especially of the subsequent reperfusion injury on the behavior and survival of *D.m*. To address this knowledge gap, we subjected two wild-type *D.m*. strains (Canton-S and Oregon-R) to severe hypoxia (<0.3% O_2_) under standardized environmental conditions in a well-constructed hypoxia chamber. During posthypoxic reperfusion (21% O_2_), we assessed fly activity (evoked and spontaneous) and analyzed molecular characteristics (oxidative stress marker abundance, reactive oxygen species (ROS) production, and metabolic activity) at various timepoints during reperfusion. First, we established standard conditions to induce hypoxia in *D.m*. to guarantee stable and reproducible experiments. Exposure to severe hypoxia under defined conditions impaired the climbing ability and reduced the overall activity of both *D.m*. strains. Furthermore, a majority of the flies died during the early reperfusion phase (up to 24 h). Interestingly, the flies that died early exhibited elevated activity before death compared to that of the flies that survived the entire reperfusion period. Additionally, we detected increases in ROS and stress marker (Catalase, Superoxide Dismutase and Heat Shock Protein 70) levels as well as reductions in metabolic activity in the reperfusion phase. Finally, we found that changes in environmental conditions impacted the mortality rate. In particular, decreasing the temperature during hypoxia or the reperfusion phase displayed a protective effect. In conclusion, our data suggest that reperfusion-dependent death might be associated with elevated temperatures, predeath activity, and oxidative stress.

## Stroke: reperfusion injury model in fruit flies

A new fruit fly model of reperfusion injury, the tissue damage commonly seen in stroke that occurs when an organ is starved of oxygen and then exposed to oxygen again, opens new possibilities for understanding stroke pathology. Pardes Habib from Aachen University, Germany, and colleagues subjected flies to extremely low oxygen levels for up to six hours before returning them to a normal oxygen environment. Most flies died after re-exposure to oxygen, but not before showing a ‘last gasp’ burst of physical activity compared to individuals that survived the treatment. The return to normal oxygen levels led to an increase in signs of cellular stress and reduced metabolic activity, especially among those flies kept at warmer temperatures. The findings lay the groundwork for better understanding of reperfusion injury and how to treat it.

## Introduction

Hypoxia (low oxygen) plays crucial roles in the etiology and pathophysiology of several diseases, including obstructive sleep apnea, chronic obstructive pulmonary disease (COPD), pulmonary hypertension, heart attack (cardiac arrest/ischemia), and cerebral ischemia^1^. The latter is one of the main causes of death and disability in adults worldwide^2^. Although recent advances in reperfusion modalities after stroke, especially in endovascular stroke treatment (EST), have significantly improved clinical outcomes, only a small number of stroke patients actually benefit from these therapies^3^. Despite the high effectiveness of EST, there is a high mortality rate (29–67 %), and many stroke patients remain severely disabled; functional independence is achieved in fewer than 50% of cases after the initial 90 days^4–6^. Postischemic reperfusion injury is considered a key mechanism for the devastating clinical outcomes. One of the main causes of postischemic reperfusion injury is free radical damage due to oxidative stress^7^. Thus, there is a pressing need for more basic research to understand and develop effective neuroprotective/neurorestorative strategies addressing pathomechanisms such as oxidative stress during the reperfusion period after ischemic stroke.

*Drosophila melanogaster* (*D.m*.) is a suitable model for analysis of hypoxia-related detrimental effects and elucidation of potential protective agents and biological mechanisms for stroke treatment. The major advantages of *D.m*. are its short life cycle, its large numbers of progeny, its limited need for resources, and the remarkably high degree of conservation between the *D.m*. genome and the human genome with regard to many biological processes. Approximately 75% of human disease-associated genes and the entire hypoxia-induced cascade are conserved in flies^8^. The availability of various mutants, transgenic strains, and genetic manipulation techniques facilitates the study of neurodegenerative diseases, including stroke, and translation of the discoveries into humans^8–10^.

However, little is known about the impact of hypoxia and especially that of the subsequent reperfusion injury on the mortality and behavior of *Drosophila*. Moreover, there is a lack of established standard operating procedures for the induction of hypoxia in *D.m*. to guarantee valid and reproducible experimental conditions.

Our literature search (on NCBI and Web of Science) revealed 154 publications addressing hypoxia in *D.m*. but hypoxia experiments with flies were conducted only in 88 publications (Supplementary Table 1). However, in most of these reports, the impacts of environmental parameters, such as temperature, humidity, and pressure, on survival and outcome were neglected or not discussed. Approximately 31% of these 88 publications reported temperature monitoring during hypoxia experiments. While 24% used at least some sort of humidifier, only 3.4% actually stated a measurement of humidity in their experimental setups. Interestingly, none of the mentioned publications described any monitoring of pressure levels during hypoxia experiments (Supplementary Table 1).

However, some of the aforementioned environmental conditions are known to have considerable influences on the survival of stroke patients. It is widely accepted, for instance, that therapeutic hypothermia has cytoprotective and neuroprotective effects^11^.

Hence, we aimed to establish a standardized hypoxia and reperfusion protocol utilizing a self-constructed hypoxia chamber with the ability to measure and control environmental conditions such as oxygen levels, temperature, humidity, and pressure. To assess the impacts of hypoxia and reperfusion, we subjected the two commonly used wild-type *D.m*. strains (Canton-S and Oregon-R) to different durations of hypoxia followed by reperfusion for various durations and performed behavioral assessments utilizing a computer-assisted DAM system; a climbing assay (negative geotaxis); and molecular analyses of oxidative stress marker gene and protein expression, ROS production, and metabolic activity. To clarify whether the observed death occurs predominantly during hypoxia or in the reperfusion phase and to evaluate the influences of environmental conditions, we varied the latter individually or in combination and detected the resulting mortality.

We were able to demonstrate that severe hypoxia impairs the climbing ability and activity of both *Drosophila melanogaster* strains. Furthermore, the majority of the flies did not die directly from hypoxia but rather died within 24 h of reperfusion. Moreover, the latter also displayed a higher activity rate than the flies that survived the entire observation period (120 h) after hypoxia. Low humidity and high pressure led to an increased mortality rate, whereas decreasing the temperature during hypoxia and the reperfusion phase revealed a protective effect. Additionally, we provide evidence that reperfusion-dependent death might be associated with increased oxidative stress and decreased metabolic activity.

## Materials and methods

### Animals: *Drosophila melanogaster*

The *D.m*. wild-type strains Canton-S (#64349) and Oregon-R (#5) were obtained from the Bloomington *Drosophila* Stock Center (Bloomington, IN, USA). The *w;;ubi-EGFP-ODD/ubi-mRFP-nls* stock was a gift from Professor Dr. Stefan Luschnig (Institute of Neurobiology, University of Münster, Münster, Germany). All flies were raised and maintained in plastic vials containing standard cornmeal food at 25 °C under a 12 h/12 h light/dark cycle. Every five days, the flies were transferred to new vials with fresh food.

### Hypoxia chamber

Hypoxia was induced with nitrogen (N_2_) in a self-constructed hypoxia chamber consisting of a heated water chamber, a gas-flow regulator, a humidifier and an airtight acrylic glass compartment with a stainless-steel bottom plate and lid (Supplemental Fig. 1 a, b). The chamber enabled the control and monitoring of oxygen levels (Greisinger GOX 100 T O_2_ – Sensor), humidity, temperature (Habor Thermo-Hygrometer) and pressure (Fluke 700RG06 100 PSIG pressure gauge).

### Mortality rate

To study the impact of hypoxia on survival, male Canton-S and Oregon-R flies at 1 to 5 days of age were subjected to 1 to 6 h of severe hypoxia (<0.3% O_2_). The mortality rate was assessed daily for the following 5 days under normoxic conditions. The temperature, pressure, and humidity inside the chamber were recorded regularly during this time (Supplemental Fig. 1b). In initial experiments, we detected sex-dependent vulnerability to hypoxia/reperfusion injury. Females seemed to exhibit greater resistance to the detrimental effects of hypoxia. The reasons for these sex differences are unknown so far. Since we aimed to standardize our procedure as much as possible, all experiments were conducted with male flies only. Each experiment was repeated at least 4 times for the examined hypoxia period. Three vials containing 20 male flies for each genotype (a total of 60 flies per experiment) were subjected to hypoxia once. After establishment of the half-lethal hypoxia duration, the following experiments were conducted utilizing 2.5 h of severe hypoxia (<0.3% O_2_).

### *Drosophila* activity monitoring (DAM) assay

To evaluate activity after hypoxia, wild-type flies were transferred into a *Drosophila Activity Monitoring* system (Model DAM2, Trikinetics, Inc., USA). Each fly was placed individually into a polycarbonate vial containing food (2% agar and 4% sucrose). Activity was monitored by recording the light beam interruptions caused by the fly passing through the light beam in the center of each vial. The flies were kept in the DAM system for a total of 5 days, during which the numbers of interruptions of the light beams were recorded every hour. The experiment was conducted 3 times in total, including 180 flies for each genotype and treatment.

### Negative geotaxis assay

A negative geotaxis assay was conducted as described previously^12^ with slight modifications. Climbing ability was assessed 3, 6, 24, 48, 72, 96, and 120 h after hypoxia, and each vial contained a group of 20 male flies.

In 4 individual experiments, the negative geotaxis performance was evaluated for a total of 240 flies for each genotype. The impact of hypoxia on negative geotaxis was assessed using only living flies. The dead flies were removed, and the results are therefore presented as mortality-adjusted percentages.

### Immunoblot analysis

Flies were snap-frozen in liquid nitrogen, and their heads were collected and homogenized in RIPA buffer (10 µl/head) using a Speedmill P12 (Analytik Jena AG). The total protein concentration was measured with a DC^TM^ Protein Assay Kit (Bio-Rad Laboratories, USA). Twenty micrograms of protein was separated via SDS-PAGE before being blotted onto nitrocellulose membranes. The membranes were blocked with 5% skim milk in TBS-T, after which they were incubated with the primary antibodies overnight at 4 °C. Incubation with the corresponding HRP-conjugated secondary antibody was carried out for 2 h at room temperature and followed by signal detection via chemiluminescence (SuperSignal^TM^ West Femto Maximum Sensitivity Substrate, Thermo Scientific, Rockford, USA). The following antibodies were used: mouse anti-GFP (1:1000 #11814460001, Roche Diagnostics) and mouse anti-*Drosophila* CSP-2 (1:500, #6D6, Developmental Studies Hybridoma Bank, IA USA).

### RT-qPCR

Gene expression analyses were performed with tissue from fly heads after hypoxia or the corresponding normoxia. After homogenizing the snap-frozen fly heads in PeqGold^TM^ (PeqLab #30-2010, Erlangen, Germany), total RNA was prepared by phenol-chloroform extraction as previously described^13^. Complementary DNA was synthesized using an iScript^TM^ cDNA Synthesis Kit (Bio-Rad Laboratories, CA, USA) and random hexanucleotide primers (Invitrogen, Karlsruhe, Germany) using 1 µg of total RNA according to the manufacturer´s protocol.

RNase-free H_2_O (Merck, 64293, Darmstadt, Germany) served as the no-template control (NTC). RT-qPCR analysis was performed using a MyIQ^TM^ RT-qPCR detection system (Bio-Rad Laboratories, CA, USA). The expression levels of the target genes and two housekeeping genes, *Actin-5C* (*Act5c*) and *Elongation factor 1-alpha 2* (*eEF1alpha2*), were measured as cycle threshold (Ct) values, and relative quantification was performed by the ΔΔCt method using qbase^TM^ + software (Biogazelle, Gent, Belgium). The data are expressed as the expression levels of the target genes relative to those of *Act5c* and *eEF1alpha2*. Forward (fwd) and reverse (rev) primers were used for the following genes (5′→3′): *Act5c* (fwd: TTT CAA ACC GTG CGG TCG CT, rev: CAT CAC ACC CTG GTG ACG GG), *eEF1alpha2* (fwd: GCG TGG GTT TGT GAT CAG TT, rev: GAT CTT CTC CTT GCC CAT CC), *Similar* (*Sima*) (fwd: TTT GCC ATT GAA AAC CGA CGA, rev: CTT GAG GAA AGC GAT GGT GAT), *Tango* (fwd: CTT GAG GAA AGC GAT GGT GAT, rev: CCG GAC AAG CTC ACC ATT CT), *Lactate dehydrogenase* (*Ldh*) (fwd: CAG TTC GCA ACG AAC GCG CA; rev: CAG CTC GCC CTG CAG CTT GT), *inducible nitric oxide synthase* (*iNOS*) (fwd: AAC GTT CGA CAA ATG CGC CAA, rev: GGA TGG TCC ACT TCA TGG CT), *Catalase* (fwd: ACC AGG GCA TCA AGA ATC TG, rev: AAC TTC TTG GCC TGC TCG TA), *Heat shock protein 70* (*Hsp70)* (fwd: GCT GAC GTT CAG GAT TCC AT, rev: CGG AGT CTC CAT TCA GGT GT), and *Superoxide dismutase* (*SOD*) (fwd: GGA GTC GGT GAT GTT GAC CT, rev: GTT CGG TGA CAA CAC CAA TG).

### Metabolic activity assay

A CellTiter-Blue^®^ Cell Viability Assay (Promega, USA) was used to assess metabolic activity after hypoxia. This test was performed as previously described^14^. In brief, 40 fly heads were homogenized using a Speedmill^TM^ P12 (Analytik Jena AG, Jena, Germany) in 1 mL of 20 mM Tris buffer, pH 7.0, and centrifuged at 1600 x *g* for 10 min at 4 °C. The supernatant was incubated with 0.2 mg/mL resazurin for 4 h. The fluorescence of the converted resazurin was measured at an excitation wavelength of 573 nm and an emission wavelength of 584 nm. The metabolic activity of Canton-S and Oregon-R was evaluated 0, 6, and 24 h after hypoxia.

### ROS assay

To determine the ROS levels after hypoxia, fly head lysates were incubated with 2’,7’-dichlorodihydrofluorescein diacetate (DCFH-DA) as previously described^14^ with slight modifications. The fly head lysates were incubated with 5 mM DCFH-DA for 30 min and centrifuged at 400 x *g* for 5 min at 4 °C. The pellet was washed once and resuspended in Tris buffer. The fluorescence of DCFH-DA was measured at an excitation wavelength of 488 nm and an emission wavelength of 525 nm. The ROS production of Canton-S and Oregon-R was evaluated 0, 6, and 24 h after hypoxia.

### Statistics

Data analysis and visualization were performed using GraphPad Prism (version 8.4.3, San Diego, CA, USA). The data for each experiment comprise the results of four independent experiments with three technical replicates, each including a total of 240 flies per group. Residuals were analyzed for normal distribution using the Shapiro–Wilk and D’Agostino-Pearson omnibus normality tests. Variance homogeneity was tested using the Bartlett test or Spearman’s rank correlation test for heteroscedasticity. For identification of outliers, a ROUT test was utilized. If the normality or homogeneity test result was significant, nonparametric tests were applied instead of one-way or two-way ANOVA. The data are given as arithmetic means ± SEMs. The level of significance was set at *p* < 0.05. Asterisks indicate significant between-group differences; “#” indicates a significant difference between hypoxia and the corresponding normoxia control. The individual data points for each experiment are given in the legends.

## Results

### Increases in the duration of severe hypoxia are associated with increased death rates in both *Drosophila melanogaster* strains

To develop a reliable, efficient, and reproducible hypoxia protocol for *Drosophila melanogaster* (*D.m.)*, we constructed a hypoxia chamber with the ability to monitor and control oxygen levels, temperature, humidity, and pressure (Supplemental Fig. 1a, b).

**Fig. 1:**
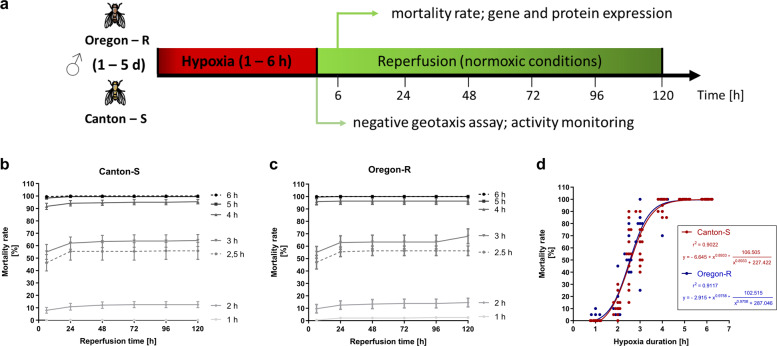
Mortality rates of Canton-S and Oregon-R *D.m*. subjected to different durations of N_2_-induced hypoxia. Schematic illustration of the hypoxia protocol in *D. melanogaster*. **a** The frequently used wild-type *D. melanogaster* strains Canton-S and Oregon-R were subjected to different durations (1–6 h) of severe hypoxia (<0.3% O_2_) followed by 120 h of reperfusion. Behavioral testing (negative geotaxis and DAM assays), mortality rate assessment, and gene and protein analyses were performed in the posthypoxic reperfusion phase. **b** Death rates of Canton-S *D.m*. and **c** Oregon-R *D.m*. after 1 to 6 h of severe hypoxia (<0.3% O_2_) followed by 120 h of reperfusion. **d** Nonlinear regression of the hypoxia duration-dependent death rates of Canton-S *D.m*. and Oregon-R *D.m*. The data are shown as the mean ± SEM from 4 independent experiments including a total of 240 male flies per genotype for each hypoxia period.

To investigate the influence of severe hypoxia (<0.3% O_2_) and subsequent reperfusion (21% O_2_) on the mortality rates of the two wild-type *D.m*. strains Canton-S and Oregon-R and to define a half-lethal hypoxic stimulus for our later experiments, we subjected both *D.m*. strains to different durations of severe N_2_-induced hypoxia under controlled environmental conditions (29 °C, 50–70% humidity and atmospheric pressure) followed by various reperfusion periods (0–120 h) (Fig. 1a). Here, increases in hypoxia durations resulted in elevations in mortality rates in both fly strains (Fig. 1b, c). Hypoxia durations of 4 to 6 h induced a death rate of 80 to 100%, while 2.5 h of hypoxia resulted in approximately 50% death. Nonlinear regression analysis of the results obtained for different hypoxia durations revealed similar posthypoxic death rates in Canton-S (*r*^2^ = 0.90) and Oregon-R (*r*^2^ = 0.91) (Fig. 1d), indicating that we established a reliable and reproducible hypoxia protocol for our experiments.

### The hypoxia response-regulating HIF/Sima-pathway is activated in *D.m*. after 2.5 h of severe hypoxia

**Fig. 2:**
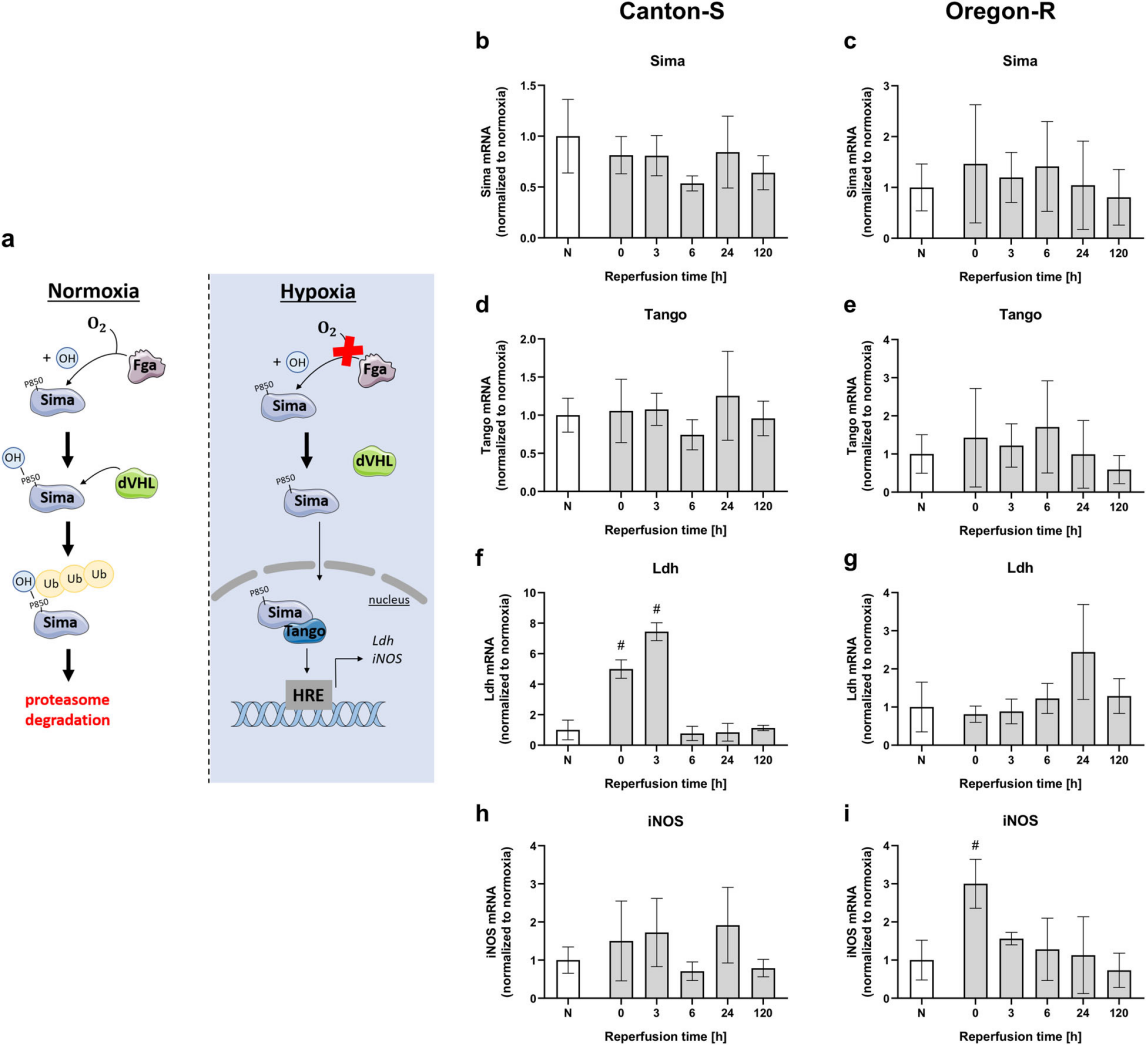
Posthypoxic mRNA levels of proteins involved in the Sima pathway in Canton-S and Oregon-R *D.m*. after exposure to 2.5 h of hypoxia. **a** Schematic illustration of the Sima cascade. mRNA levels of *Sima* (**b**, **c**), *Tango* (**d**, **e**), *Ldh* (**f**, **g**), and *iNOS* (**h**, **i**) in both fly strains were evaluated at different posthypoxic reperfusion timepoints (0, 3, 6, 24, and 120 h after hypoxia). The target gene levels are presented as ratios to the levels of the constitutive genes *Act5c* and *eEF1alpha2* and were normalized to the corresponding normoxia values. The graphs show the means ± SEMs from 3 independent experiments including 180 male flies per genotype for each treatment. Kruskal–Wallis test followed by Dunn’s multiple comparison test. *p < 0.05.

The only known homologue of the conserved master regulator of the hypoxia response HIF (hypoxia inducible factor) alpha in *Drosophila* is the bHLH/PAS domain transcription factor Similar (Sima)^15,16^. Under normoxic conditions, Sima is hydroxylated by the prolyl hydroxylase Fatiga (Fga) in its oxygen-dependent degradation (ODD) domain; subsequently, Sima is labeled for proteasomal degradation by the dVHL ubiquitin ligase (Fig. 2a)^17–19^. Upon hypoxia, the hydroxylation of Sima does not occur, resulting in Sima accumulation in the cytosol followed by translocation into the nucleus. In the nucleus, Sima dimerizes with the HIF-beta subunit homologue Tango (Tgo) and binds to the respective hypoxia response elements (HREs) to regulate the transcription of hypoxia response genes such as *Ldh* and *iNOS*^17–19^. To visualize and quantify sufficient activation of the HIF/Sima pathways after 2.5 h of hypoxia, we used a hypoxia biosensor consisting of GFP fused to the ODD domain of Sima (w;;ubi-EGFP-ODD/ubi-mRFP-nls), as recently described by Misra and colleagues^16^. We detected increases in EGFP-ODD protein levels after 2.5 h of hypoxia that were further augmented over the course of 24 h of reperfusion (Supplemental Fig. 2a, b). Hyperoxia (60% O_2_) resulted in overall reductions in EGFP-ODD protein signals in Western blot analysis (Supplemental Fig. 2c, d). The wild-type strains displayed no evidence for regulation of *Sima* mRNA levels (Fig. 2a, b) or *Tango* mRNA levels (Fig. 2c, d) after 2.5 h of hypoxia followed by 120 h of reperfusion. In the initial posthypoxic phase, the mRNA levels of the *HIF/Sima* downstream targets *Ldh* (Fig. 2e, f) and *iNOS* (Fig. 2g, h) were upregulated, but they normalized during the course of reperfusion. Thus, our results show activation of the Hif1a/Sima cascade after hypoxia.

### Hypoxia impairs climbing ability and activity within the reperfusion period of 5 days

**Fig. 3:**
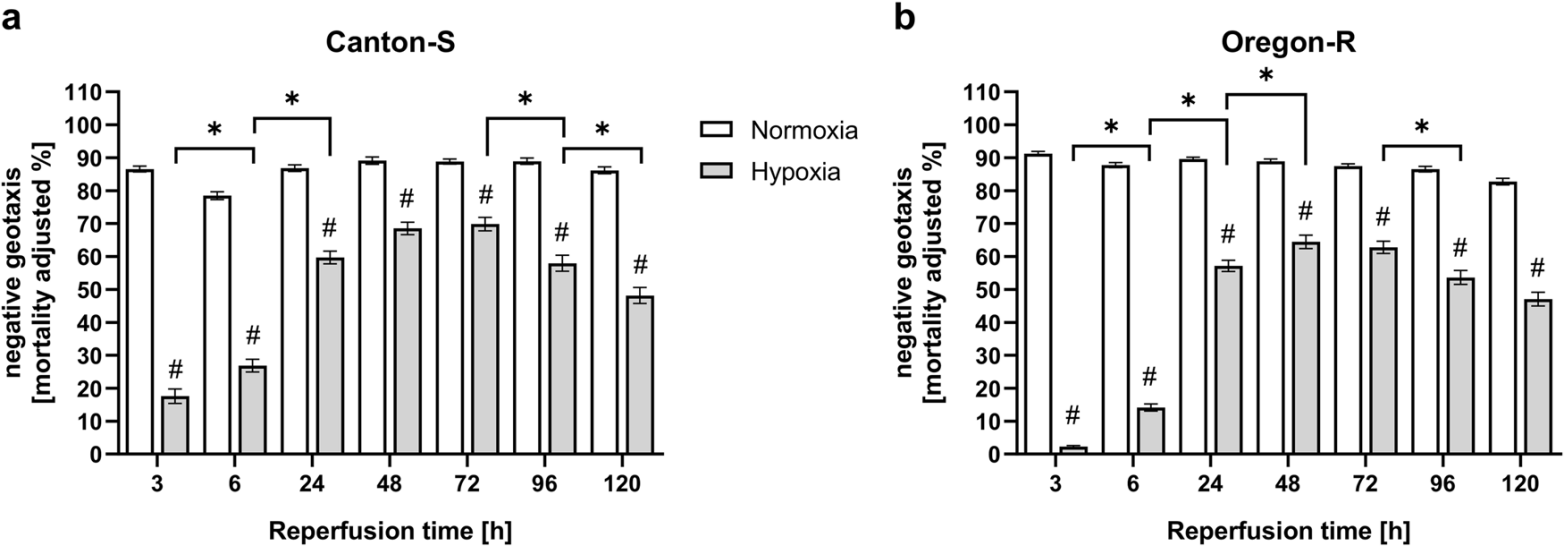
Mortality-adjusted negative geotaxis/climbing ability of Canton-S and Oregon-R *D.m*. subjected to 2.5 h of N_2_-induced hypoxia followed by 120 h of reperfusion. **a** Climbing ability of Canton-S *D.m*. and **b** Oregon-R *D.m*. after 2.5 h of severe hypoxia (<0.3% O_2_) or normoxia (21% O_2_) followed by 120 h of reperfusion. Negative geotaxis was assessed at various timepoints (3, 6, 24, 48, 72, 96, and 120 h after hypoxia/normoxia). Groups of 20 flies were transferred into empty plastic vials and tapped to the bottom. The percentage of live flies capable of reaching the 8 cm mark in 10 s was recorded. The graphs show the means ± SEMs from 4 independent experiments including 240 male flies per genotype for each treatment. Two-way ANOVA followed by Bonferroni’s multiple comparison test. #*p* < 0.001. # indicates significance compared to the corresponding normoxia-exposed flies. For comparison of hypoxia with the previous reperfusion timepoint, one-way ANOVA followed by Tukey’s multiple comparison test was used. *p < 0.05.

To evaluate the influence of hypoxia and particularly posthypoxic reperfusion on the behavior and outcomes of the two wild-type fly strains, we analyzed climbing ability (negative geotaxis) and activity at various time points. The negative geotaxis response was significantly impaired in the hypoxia-exposed flies (# *p* < 0.001) compared to the normoxia control flies over the entire reperfusion period of 5 days. The greatest impairment of climbing ability in both fly strains was observed after 3 h of reperfusion following hypoxia. Bell-shaped recovery curves with peaks at 48 h of reperfusion (Oregon-R) and 72 h of reperfusion (Canton-S) were evident (Fig. 3a, b).

**Fig. 4:**
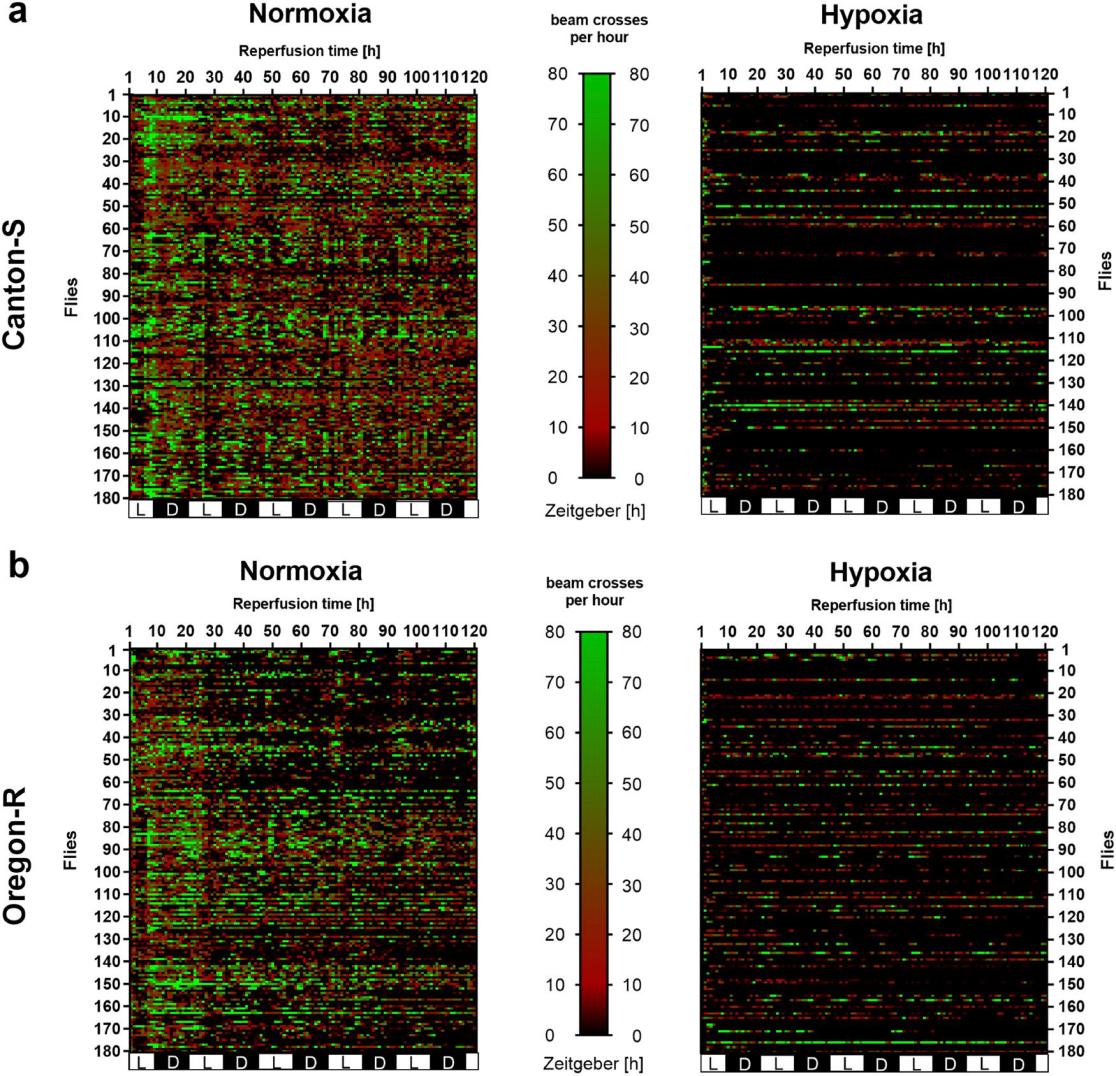
Activity of Canton-S and Oregon-R *D.m*. after 2.5 h of N_2_-induced hypoxia. **a** Heatmap displaying the activity of Canton-S *D.m*. and **b** Oregon-R *D.m*. after 2.5 h of severe hypoxia (<0.3% O_2_) or normoxia (21% O_2_). Each cell shows the absolute value of beam crosses per hour, which is represented by color (black: little to no activity, red: moderate activity, green: high activity), as indicated in the legend. Flies were transferred into the DAM system immediately after being subjected to hypoxia/normoxia, and the activity was recorded for the following 120 h. Data were obtained from 3 independent experiments including a total of 180 male flies per genotype for each treatment.

Along with climbing ability, hypoxia and posthypoxic reperfusion also had decisive effects on the general activity of both fly strains. To detect and quantify these effects, we monitored the beam crosses per hour after hypoxia for 5 days using the DAM system. Both Cantons-S and Oregon-R showed significant reductions in beam crosses (activity) after hypoxia than after exposure to the corresponding normoxia conditions over the whole observation period of 120 h (Fig. 4a, b).

Based on our previous observations, we knew that the deaths of flies of both strains after hypoxia occurred primarily during the first 24 h of reperfusion. Hence, we aimed to investigate how many flies died directly after hypoxia, how many survived and how many survived after hypoxia but died during the first 24 h. Furthermore, we compared the activity of the flies that survived the 24 h after hypoxia with that of the flies that survived hypoxia but died during the 24 h of reperfusion.

**Fig. 5:**
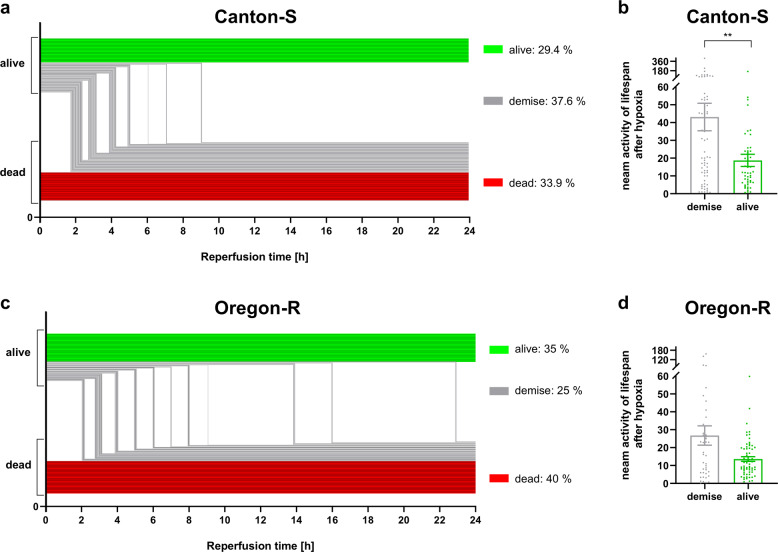
Impact of hypoxia and the posthypoxic reperfusion phase on the death rates and activity of Canton-S and Oregon-R *D.m*. Quantification of survival, instant death, and reperfusion-dependent death of **a** Canton-S *D.m*. and **c** Oregon-R *D.m*. in the first 24 h following 2.5 h of severe hypoxia (<0.3% O_2_). The posthypoxic mean activity per lifespan of **b** Canton-S *D.m*. and **d** Oregon-R *D.m*. during the first 12 h is shown. Mann–Whitney U test. *p < 0.05, **p < 0.01. The data shown were obtained from 3 independent experiments including a total of 180 male flies per genotype for each treatment.

For Canton-S, following 2.5 h of hypoxia, 29.4% of the flies survived the 24 h of reperfusion (“alive” group, in green), 37.6% survived hypoxia but died within 24 h of reperfusion (“demise” group, in gray), and 33.9% never recovered from hypoxia (“dead” group, in red). Interestingly, in the demise group of flies, we observed that the animals died within the first 12 h after hypoxia (Fig. 5a). In contrast, more Oregon-R animals appeared to die directly after hypoxia (dead group: 40%) and to survive the entire 24 h of reperfusion (alive group: 35 %) after hypoxia than Canton-S flies. However, the Oregon-R demise group was smaller (25%) than the Canton-S group, and the animals in this group survived longer than those in the Canton-S group (Fig. 5b). Another difference between the two demise groups was the mean lifespan activity after hypoxia. While the Canton-S demise group showed significantly (**p < 0.01) greater activity than the alive group (Fig. 5c), Oregon-R did not display a statistically significant difference between groups (Fig. 5d).

These data suggest that the majority of the flies did not die directly from hypoxia but rather within the first 24 h of reperfusion. Furthermore, the flies that died within 24 h of reperfusion also showed higher predeath activity than the flies that survive the entire observation period after hypoxia.

### Hypoxia increases ROS production and the expression of antioxidative stress-related proteins within 24 h of reperfusion

**Fig. 6:**
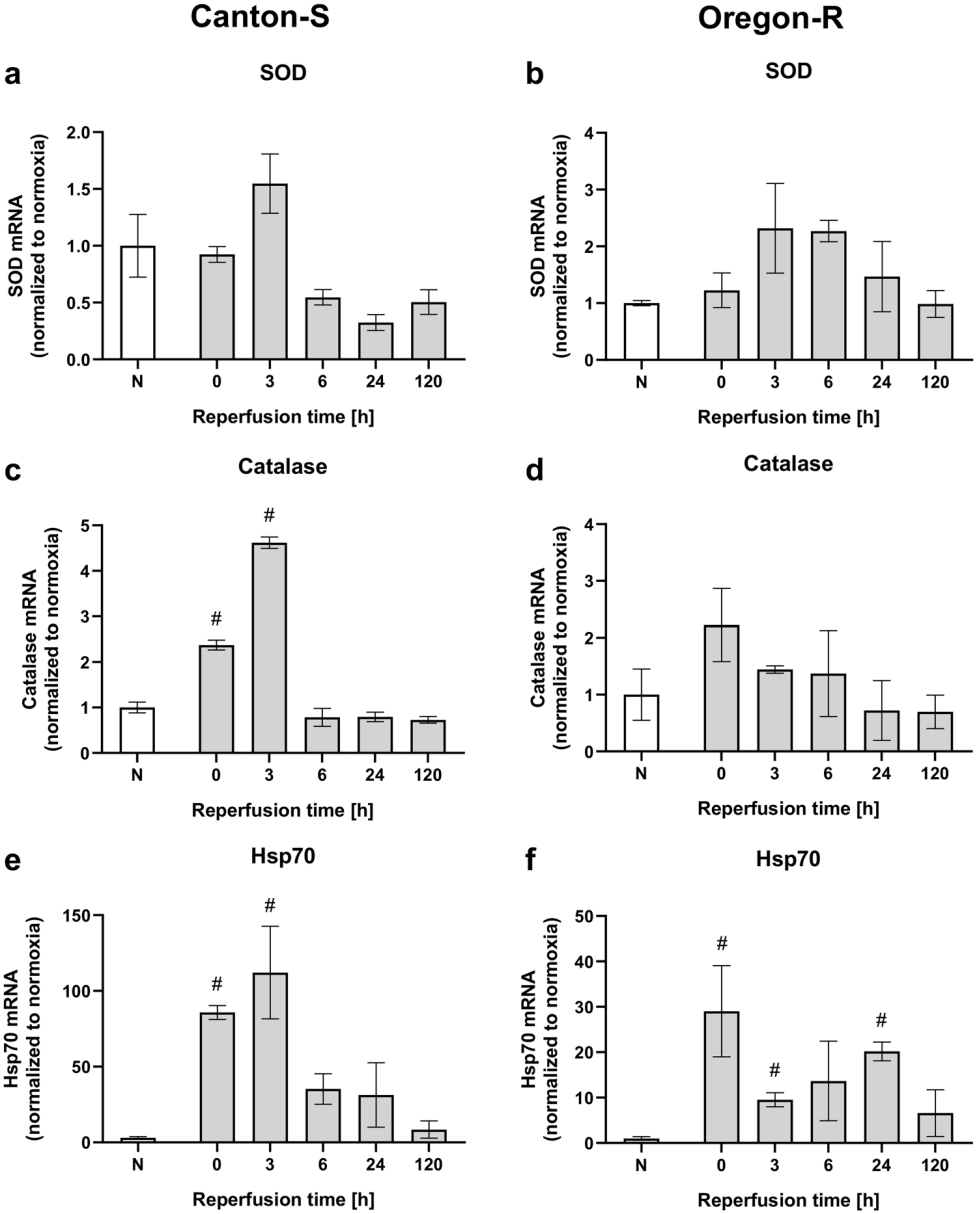
Posthypoxic mRNA levels of antioxidant proteins (*SOD*, *Catalase*, *Hsp70*) in Canton-S and Oregon-R *D.m*. subjected to 2.5 h of hypoxia. The mRNA levels of *SOD* (**a**, **b**), *Catalase* (**c**, **d**), and *Hsp70* (**e**, **f**) in both fly strains were evaluated at different posthypoxic reperfusion timepoints (0, 3, 6, 24, and 120 h after hypoxia). The target gene levels are presented as ratios to the levels of the constitutive genes *Act5c* and *eEF1alpha2* and were normalized to the levels in the corresponding normoxia-exposed flies. The graphs present the means ± SEMs from 3 independent experiments including 180 male flies per genotype for each treatment. Kruskal–Wallis test followed by Dunn’s multiple comparison test. *p < 0.05.

**Fig. 7:**
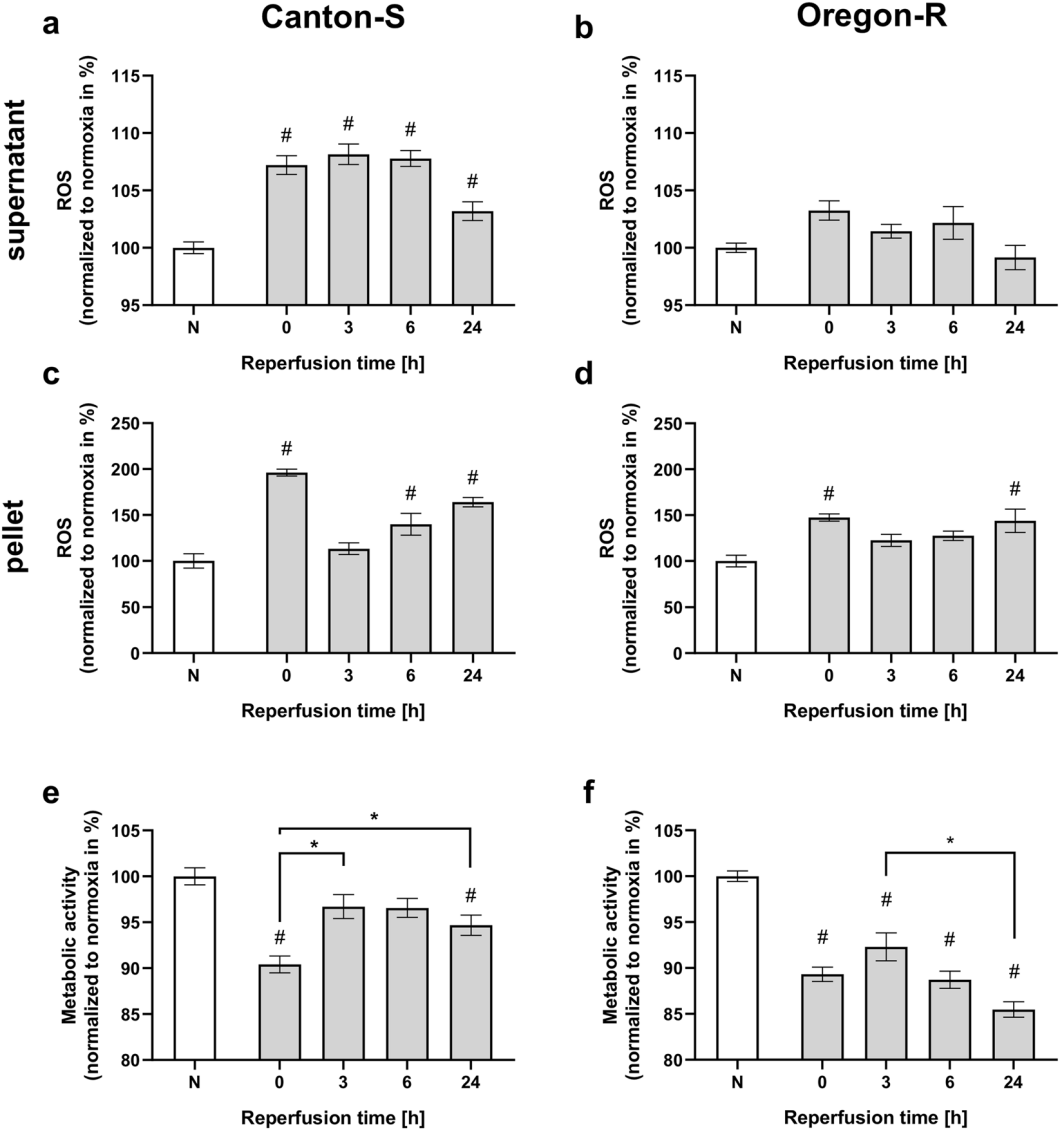
Posthypoxic ROS production and metabolic activity of Canton-S and Oregon-R *D.m*. during a reperfusion period of 24 h. ROS production was measured via DCF-DA fluorescence at various reperfusion timepoints (0, 3, 6, and 24 h) after 2.5 h of severe hypoxia. ROS production in the supernatant is shown in **a** and **b**, and ROS in the pellet is depicted in **c** and **d**. In parallel, metabolic activity (**e**, **f**) was assessed using a Cell Titer Blue assay. The graphs present the means ± SEMs of 4 independent experiments. Kruskal–Wallis test followed by Dunn’s multiple comparison test. *p < 0.05, # indicates significance compared to normoxia.

Postischemic oxidative stress is assumed to be caused by an imbalance between reactive oxygen species (ROS) levels and the activity of antioxidant defense system proteins, including the enzymes Catalase and Superoxide dismutase (SOD), and Heat shock protein 70 (Hsp70). This oxidative stress is considered to be a major contributor to death. To investigate this stress in flies, we first examined the mRNA levels of *SOD*, *Catalase*, and *Hsp70* in the heads of flies of both wild-type fly strains after 2.5 h of severe hypoxia followed by a reperfusion period of 120 h. Both strains showed a tendency for *SOD* mRNA levels to increase in the initial phase after hypoxia, but the levels decreased over the course of reperfusion (Fig. 6a, b). In contrast, the mRNA levels of *Catalase* were significantly increased in Canton-S immediately after hypoxia and after three hours of reperfusion (*p* < 0.01), whereas this tendency was not statistically significant in Oregon-R (Fig. 6c, d). In the case of *Hsp70*, significant increases in mRNA levels were observed in both strains after hypoxia, and the largest increases in *Hsp70* mRNA levels were registered in the initial phase after hypoxia (Fig. 6e, f). Next, we measured ROS production in head homogenates (pellets) and in the supernatant after hypoxia followed by various durations of reperfusion. In the supernatant of Canton-S flies, a significant posthypoxic increase in ROS within the reperfusion period of 24 h was detected (Fig. 7a). However, such an increase in ROS was not evident in the supernatant of Oregon-R flies (Fig. 7b). The pellet contained significantly higher amounts of ROS than the supernatant for both strains, particularly directly after hypoxia and after a reperfusion period of 24 h (Fig. 7c, d). Metabolic activity was decreased in Canton-S after hypoxia and recovered during reperfusion for 24 h (Fig. 7e). In contrast, the metabolic activity in Oregon-R remained reduced during the whole reperfusion period in the hypoxia-exposed flies compared to the corresponding normoxia control flies (Fig. 7f). These results suggest that increases in oxidative stress and reductions in metabolic activity occurred in both strains of wild-type flies, especially in the initial phase after hypoxia. This might be a possible explanation for the observed mortality immediately after hypoxia.

### Environmental conditions, particularly temperature, influence survival during and after hypoxia

**Fig. 8:**
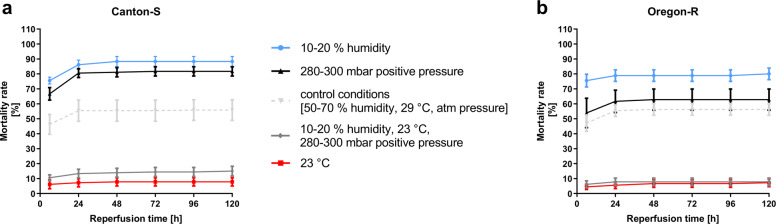
Impacts of environmental conditions on posthypoxic survival rates of Canton-S and Oregon-R *D.m*. subjected to 2.5 h of N_2_-induced hypoxia. **a** Death rates of Canton-S *D.m*. and **b** Oregon-R *D.m*. after 2.5 h of severe hypoxia (<0.3% O_2_) upon modification of humidity, temperature or pressure or a combination of all three parameters during hypoxia compared to those under standardized control conditions (in gray). The data points represent the means ± SEMs of 4 independent experiments (control conditions are in gray) including a total of 240 male flies or *n* = 3 independent experiments including a total of 180 male flies per genotype for each hypoxia period.

**Fig. 9:**
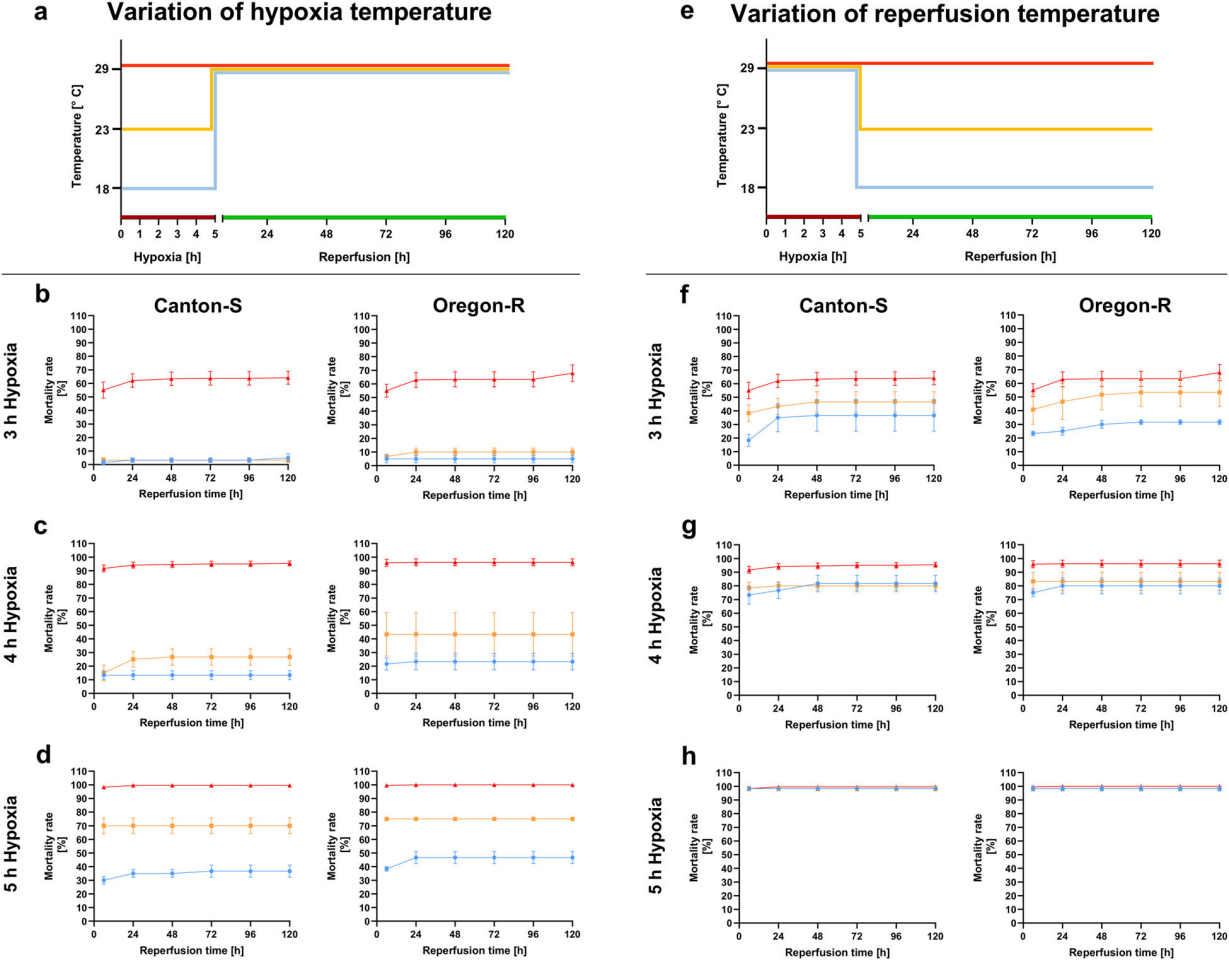
Impacts of different reperfusion and hypoxia temperatures on the mortality rates of Canton-S and Oregon-R *D.m*. **a** Schematic illustration of the experimental procedure used to assess the effects of different hypoxia temperatures on survival. Hypoxia of different durations (3–5 h) was performed at 18 °C, 23 °C, and 29 °C followed by 120 h of reperfusion at 29 °C. The death rates of Canton-S *D.m*. and Oregon-R *D.m*. after **b** 3 h, **c** 4 h, and **d** 5 h of severe hypoxia (<0.3% O_2_) at 18 °C, 23 °C, and 29 °C followed by 120 h of reperfusion were determined. **e** Schematic illustration of the experimental procedure. In brief, hypoxia was performed at 29 °C for 3 to 5 h followed by 120 h of reperfusion at 18 °C, 23 °C, and 29 °C. The death rates of Canton-S *D.m*. and Oregon-R *D.m*. after **f** 3 h, **g** 4 h, and **h** 5 h of severe hypoxia (<0.3% O_2_) followed by 120 h of reperfusion at 18 °C, 23 °C, and 29 °C were determined. The data are shown as the means ± SEMs of 3 independent experiments including a total of 60 male flies per genotype for each hypoxia duration and temperature.

As already mentioned in the introduction, according to our research, very few published studies assessing the influence of hypoxia on *D.m*. have monitored or controlled environmental conditions. Overall, there are few data on the influences of humidity, temperature, and pressure on the survival of animals after hypoxia. Therefore, we introduced both wild-type fly strains to hypoxia with altered environmental conditions individually or in combination and observed their survival over 5 days. We observed that a decrease in humidity (10–20%) resulted in 20–30% higher mortality after hypoxia than the control condition (50–70% humidity, 29 °C, atmospheric pressure) (Fig. 8a, b). Similarly, an increase in atmospheric pressure by 280 to 300 mbar increased the rate of posthypoxic death by 10–20%. Strikingly, compared to the control condition, a reduced temperature of 23 °C during hypoxia reduced the mortality of both fly strains by 50%. The temperature effect appeared to have a large impact on survival, since the low temperature during hypoxia (23 °C) still led to a 40–50% reduction in posthypoxic mortality despite the combination with increased pressure (280 to 300 mbar) and decreased humidity (10–20%). To study the effect of temperature on posthypoxic mortality in more detail, we exposed flies to reduced temperatures during hypoxia of 23 °C and 18 °C in addition to control conditions at 29 °C. During reperfusion, all flies were maintained under the same control conditions (50–70% humidity, 29 °C, atmospheric pressure) (Fig. 9a). A hypoxia duration of 3 h at 29 °C led to mortality rates of 60–70% in both *D.m*. strains, while a reduction in the temperature to 23 or 18 °C caused more than 50% reductions in mortality (Fig. 9b). An increase in the hypoxia duration to 4 h at 29 °C resulted in a death rate of approximately 90% in both wild-type fly strains, while the death rates at 23 °C ranged from 20 to 50%. A temperature of 18 °C during hypoxia resulted in mortality below 30% after 5 days of reperfusion (Fig. 9c). The lowest temperature resulted in a mortality rate of less than 50% even after 5 h of hypoxia, whereas the higher temperatures (23 and 29 °C) resulted in mortality rates of 70 to 100% (Fig. 9d).

Subsequently, we aimed to investigate whether a reduction in the ambient temperature from 29 °C to 23 or 18 °C during the reperfusion period has a similar protective effect as a reduction in temperature during hypoxia. For this purpose, we subjected both wild-type strains to a hypoxia period of 3 to 5 h under standard conditions (50–70% humidity, 29 °C, atmospheric pressure). For the reperfusion period of 5 days, we either maintained the standard conditions or reduced the temperature to 23 or 18 °C (Fig. 9e). After 3 h of hypoxia, the decreased temperatures had significantly reduced mortality, resulting in death rates below 50%, for both *D.m*. strains (Fig. 9f). Compared to a temperature of 29 °C, low temperatures (23 and 18 °C) had protective effects over a hypoxia duration of 4 h, but the two low temperatures did not seem to differ in their effects (Fig. 9g). With a further increase in the hypoxia duration to 5 h, the different temperatures in the reperfusion phase did not seem to play relevant roles, since nearly all flies died after this stimulus (Fig. 9h).

In conclusion, environmental conditions, such as humidity, temperature, and pressure, have effects on the mortality of *D.m*., stressing the need to monitor/control these conditions to achieve reproducible results. Furthermore, low temperature seems to have a very strong protective effect after hypoxia. Interestingly, the observed protective effect of temperature reduction at the beginning and during hypoxia was greater than that of temperature reduction during the reperfusion phase.

### Low temperatures during hypoxia mitigate increased ROS production and expression of antioxidative stress-related proteins after hypoxia

**Fig. 10:**
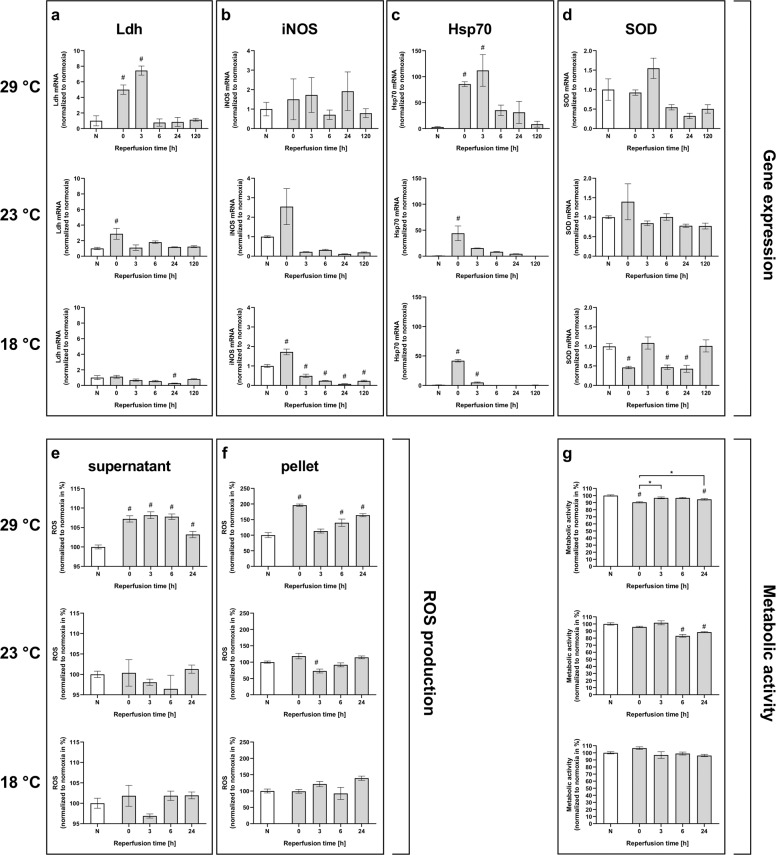
Impacts of different temperatures during hypoxia on antioxidant protein expression, ROS production, and metabolic activity. The mRNA levels of *Ldh* (**a**), *iNOS* (**b**), *Hsp70* (**c**), and *SOD* (**d**) following severe hypoxia at 29 °C, 23 °C, and 18 °C were evaluated at different reperfusion timepoints (0, 3, 6, 24, and 120 h after hypoxia). The target gene levels are presented as ratios to the levels of the constitutive gene *Act5c* and normalized to the levels in the corresponding normoxia-exposed flies. The graphs present the means ± SEMs from 3 independent experiments including 180 male flies per genotype for each treatment. Kruskal–Wallis test followed by Dunn’s multiple comparison test. *p < 0.05. ROS production in the supernatant (**e**) and in the pellet (**f**) following 2.5 h of severe hypoxia at 29 °C, 23 °C, and 18 °C was measured at various reperfusion timepoints (0, 3, 6, and 24 h) via DCF-DA fluorescence. The metabolic activity after 2.5 h of severe hypoxia at 29 °C, 23 °C, and 18 °C (**g**) was assessed using a Cell Titer Blue assay. The graphs present the means ± SEMs of 4 independent experiments. Kruskal–Wallis test followed by Dunn’s multiple comparison test. *p < 0.05, # indicates significance compared to normoxia.

After discovering that the greatest protective effects were achieved via reduction of the temperature during hypoxia, we aimed to further investigate the impacts of low temperatures during hypoxia on Sima pathway markers, antioxidative stress markers, ROS production, and metabolic activity. We analyzed the posthypoxic mRNA levels of *Ldh*, *iNOS*, *Hsp70*, and *SOD* in Canton-S flies after 2.5 h of hypoxia at 23 °C and 18 °C with a reperfusion temperature of 29 °C. *Ldh* and *iNOS* mRNA levels were upregulated in the initial posthypoxic phase (at 0 h of reperfusion) following hypoxia at 23 °C. Notably, the hypoxia-dependent increases in *Ldh* mRNA levels were lower at reduced temperatures than at 29 °C. While *iNOS* mRNA levels were also increased in the initial posthypoxic phase after hypoxia at 18 °C, *Ldh* mRNA levels after hypoxia were not significantly different from those in the normoxia control flies (Fig. 10a, b).

*Hsp70* mRNA levels were markedly increased at 0 h of reperfusion after hypoxia at both 23 °C and 18 °C. Notably, the upregulation of *Hsp70* mRNA levels declined with decreasing hypoxia temperature. Similarly, *SOD* mRNA levels were also upregulated at 0 h of reperfusion following hypoxia at 23 °C but were not significantly higher than those in the corresponding normoxia control flies after hypoxia at 18 °C (Fig. 10c, d).

Moreover, in contrast to the flies subjected to hypoxia at 29 °C, the flies subjected to reduced temperatures of 23 °C and 18 °C displayed no significant increases in ROS production compared to the corresponding normoxia control flies (Fig. 10e, f).

Metabolic activity decreased in the late posthypoxic reperfusion phase (6–24 h) after hypoxia at 23 °C but was not significantly different from that in the corresponding normoxia control flies after hypoxia at 18 °C (Fig. 10g).

These results suggest that low temperatures during hypoxia mitigate hypoxia-induced increases in oxidative stress and reductions in metabolic activity. This might serve as an explanation for the protective effects observed with lower temperatures during hypoxia.

## Discussion

In this study, we analyzed the impact of severe hypoxia (<0.3% O_2_) on adult male flies of the wild-type *Drosophila melanogaster* (*D.m*.) strains Canton-S and Oregon-R. Our analysis focused on the hypoxia-induced effects within the reperfusion (21% O_2_) period of 120 h. We demonstrated that exposure to severe hypoxia under defined conditions (50–70% humidity, 29 °C, atmospheric pressure) impaired the climbing ability and reduced the overall activity of both *D.m*. strains. Interestingly, a majority of the flies died during the early phase of reperfusion (up to 24 h), and these flies exhibited greater activity before death than the flies that survived the entire observation period (120 h). Furthermore, we detected increased reactive oxygen species (ROS) levels; increased *Catalase*, *Superoxide dismutase* (*SOD*) and *Heat shock protein 70* (*Hsp70*) expression; and reduced metabolic activity in the reperfusion phase. Both low humidity and high pressure during hypoxia resulted in an enhanced mortality rate. In contrast, low temperatures, particularly during hypoxia but also during the reperfusion phase, displayed a protective effect.

Hypoxia-reperfusion injury is a crucial factor in various medical conditions, including organ transplantations, renal diseases, cardiac arrest/infarction, wound healing, and ischemic stroke^1,20,21^. In acute ischemic stroke (AIS), the timely and successful restoration of cerebral blood perfusion is currently the only approved therapeutic option. Aside from the establishment of thrombolysis with recombinant tissue plasminogen activator (rtPA), which has been implemented since 1996, seven randomized clinical trials have highlighted endovascular stroke treatment (EST) as a remarkable additional therapeutic option for patients with severe symptoms and large vessel occlusion (LVO) AIS^4–6,22–29^. The DAWN and DEFUSE-3 trials even demonstrated that EST is suitable for stroke patients with LVO up to 24 h after symptom onset, which extends the therapeutic window significantly from the narrow time window of thrombolysis, which is within 4.5 h after symptom onset^30,31^. However, despite the high recanalization rates of EST (70–80%), fewer than 50% of AIS patients achieve functional independence. In addition, poststroke mortality within 90 days remains relatively high (29–69%), even after highly effective reperfusion treatments^4–6^. To date, the cause of the high poststroke mortality rate is not fully understood, and the restricted applicability of the two abovementioned therapies for AIS patients underlines the unmet need for understanding of the pathophysiology in the reperfusion phase in order to develop promising neuroprotective therapies.

Translational gaps, especially in the field of neuroscience, are known to exist, and improper selection of models as well as improper numbers of included animals are considered to be among the causes. Thus, we identified putative biases prior to our experiments and eliminated them by establishing a standard operating procedure (Fig. 1).

Our literature research revealed that there are large variances in the applied hypoxia durations and O_2_ levels as well as the reported hypoxia tolerances of *D.m*. Fewer than one-third of the studies, for instance, monitored environmental conditions such as temperature, humidity, and pressure during hypoxia (Supplemental Table 1). Furthermore, the impact of hypoxia on the behavior, biochemistry, and survival of *D.m*. in the reperfusion phase has been poorly studied. Thus, we first utilized a custom hypoxia chamber capable of monitoring and controlling the abovementioned parameters to determine the hypoxia tolerance of the two widely used wild-type strains under defined conditions (Fig. 1 and Supplemental Fig. 1). Despite slight differences between the two strains in our analyses, the mortality rates, behavior, and overall hypoxia-dependent responses appeared to be similar, indicating the reproducible and stable conditions of our hypoxia protocol. In line with previous reports, we were able to demonstrate that *D.m*., despite being rapidly paralyzed, display a high tolerance towards hypoxia^32^. While little to no mortality was evident after one to two hours of hypoxia, three to six hours of hypoxia resulted in death rates between 60% (3 h) and 100% (6 h). Since 2.5 h of a hypoxic stimulus caused a mortality rate of approximately 50% (Fig. 1), we used 2.5 h of hypoxia for further experiments; this paradigm resulted in a sufficient impact of hypoxia and provided a reasonable number of viable flies for analysis during the reperfusion period. Activation of the hypoxia-sensing Hif/Sima cascade was confirmed by visualizing the conserved oxygen-sensing hypoxia-inducible factor 1-alpha (Hif1-alpha) homolog Sima^15^ (Supplementary Fig. 2). In line with the findings of Misra and colleagues, we were able to show the degradation of Sima with increasing durations of hyperoxia (60% O_2_) and the accumulation of Sima upon hypoxia exposure (0.3% O_2_) followed by reperfusion for various durations. In addition, we detected transcriptional upregulation of known downstream targets of the Hif-1alpha pathway, such as *Ldh* and *iNOS* (Fig. 2). This finding implies that the cell-autonomous response of *D.m*. to alterations in oxygen levels is similar to that of humans^16^. While analyzing posthypoxic locomotion ability (negative geotaxis), we observed that the hypoxia-exposed flies exhibited significantly impaired negative geotaxis compared to that of the normoxia control flies over the entire reperfusion period of 5 days. Bell-shaped recovery curves with peaks at 48 h of reperfusion (Oregon-R) and 72 h of reperfusion (Canton-S) were evident (Fig. 3). Likewise, we observed hypoxia-dependent decreases in general activity in both strains in the DAM assay. Here, the analysis of the spontaneous locomotion of individual flies after hypoxia revealed that approximately one-third of the flies did not recover from hypoxia, while approximately 66% of the flies recovered; however, half of the recovered flies died within 24 h of reperfusion (demise group) (Fig. 4). Notably, however, the demise group exhibited significantly higher activity than the corresponding group of flies that survived the entire observation period (Fig. 5). We expected a certain degree of lethargy after hypoxia in the death group but observed increased predeath activity. The question arose as to whether the increase in predeath activity occurred due to cerebral damage, for instance, or because of the evolutionary impulse of reproduction, which will be investigated in future studies. The hypothesis that patients should be physically and mentally challenged following stroke to encourage brain plasticity has long been advocated for and even implemented in some guidelines. However, early and excessive mobilization of AIS patients seems to lead to worse outcomes. The AVERT trial randomly assigned 2104 patients with ischemic or hemorrhagic stroke to either an early mobilization or usual care protocol after stroke onset and reported markedly worse outcomes after early mobilization^33^.

Ischemia-reperfusion injury is known to evoke an imbalance in the production of harmful reactive oxygen species (ROS) and endogenous antioxidant proteins^34,35^.

Hence, we analyzed the mRNA levels of antioxidant defense system proteins, including the enzymes *Catalase* and *SOD*, and *Hsp70* after various durations of reperfusion (Fig. 6). Here, hypoxia induced upregulation of the expression of the aforementioned mRNAs, particularly in the initial phase of reperfusion. Additionally, we were able to detect elevated ROS levels in the first 24 h of reperfusion in both fly strains. The elevation was accompanied by a marked decrease in metabolic activity (Fig. 7). These results might provide a reasonable explanation for the observed mortality in the initial posthypoxic phase. Consistent with this idea, increased levels of ROS have been reported in AIS patients after recombinant tissue plasminogen activator (rtPA) application^36^.

Aside from timely recanalization and its associated effects, the body temperatures of AIS patients seem to affect clinical outcomes. Therapeutic hypothermia has been reported to improve outcomes and to reduce the mortality of patients suffering from different neurological diseases, including stroke, traumatic brain injury, intracranial pressure elevation, subarachnoid hemorrhage, spinal cord injury, hepatic encephalopathy, and neonatal peripartum encephalopathy^37–39^.

Since very few of the published studies on the impact of hypoxia on *D.m*. have monitored and controlled environmental conditions, and since there are few data on the influences of humidity, temperature, and pressure on the survival of animals after hypoxia, we introduced both wild-type fly strains to hypoxia with altered environmental conditions individually or in combination and observed their survival over 5 days (Fig. 7). As expected, decreasing humidity or increasing pressure enhanced the mortality rate after hypoxia (Fig. 8). Additionally, in line with our expectations, a decrease in temperature caused a significant reduction in mortality. We then combined the detrimental humidity reduction and large pressure increase with the beneficial temperature reduction to evaluate the impacts of individual changes on mortality. Here, we were able to demonstrate that the positive effect of decreased temperature overrode the detrimental effects of increased pressure and decreased humidity. In addition, a strong effect of temperature on the mortality rate after hypoxia was expected in the poikilothermal flies. Even in homeothermic humans, increases in body temperature are known to increase infarct sizes and seem to be associated with worse prognoses after cerebral ischemia^40^. Since elevated peak body temperatures during the first days after AIS rather than on admission are known to promote infarction and poor functional outcomes of patients^41^, we aimed to compare the impact of temperature reduction during hypoxia with that of temperature reduction in the reperfusion phase. Here, lowering the temperature exerted an overall strong protective effect after hypoxia, similar to the case in humans^11^. However, the observed beneficial effects of temperature reduction at the beginning and during hypoxia were significantly greater than that of lowering the temperature during the reperfusion phase (Fig. 9). A possible explanation for the reduced mortality rate after hypoxia at low temperatures might be reductions in oxidative stress marker levels and restoration of metabolic activity (Fig. 10). We believe that environmental conditions, such as humidity, temperature, and pressure, affect the mortality of *D.m*., stressing the need to monitor/control them to achieve reproducible results. The inconsistent control of environmental conditions in several studies might explain the great variability of the reported hypoxia tolerance of *D.m*. Nitrogen (N_2_) used to supplant ambient air is usually distributed from high-pressure bottled gas and should ideally be humidified prior to administration. In addition, N_2_ should be distributed from the bottom of the hypoxia chamber to prevent constant gas flow and with accompanying dehydration of flies (see Supplementary Fig. 1).

In conclusion, we established a reliable standard operating protocol to study the impact of hypoxia-reperfusion injury in *Drosophila melanogaster*. We provide evidence that reperfusion-dependent death might be associated with elevated temperatures, predeath activity, and oxidative stress. Although there are obvious differences between flies and humans, we have shown that the molecular responses of both organisms to hypoxia are quite similar. This is highlighted not only by the fact that the oxygen-sensing HIF-1alpha pathway and its downstream cascade are conserved but also by the fact that increases in ROS, *SOD*, *Catalase*, and *Hsp70* and decreases in metabolic activity seem to occur in similar manners. Our protocol and the available genetic alteration possibilities in flies enable further investigation of hypoxia-/ischemia-dependent damage in a high number of individuals with high reproducibility and reliability.

## Supplementary information

Supplemental Material
