# Supplementary material for: Posthypoxic behavioral impairment and mortality of Drosophila melanogaster are associated with high temperatures, enhanced predeath activity and oxidative stress
Source: Exp Mol Med. 2021 Feb 9;53(2):264–80. doi: 10.1038/s12276-021-00565-3 (PMC8080651; doi:10.1038/s12276-021-00565-3)
Supplement: Supplementary file 1 — Supplemental Material [file 12276_2021_565_MOESM1_ESM.pdf]

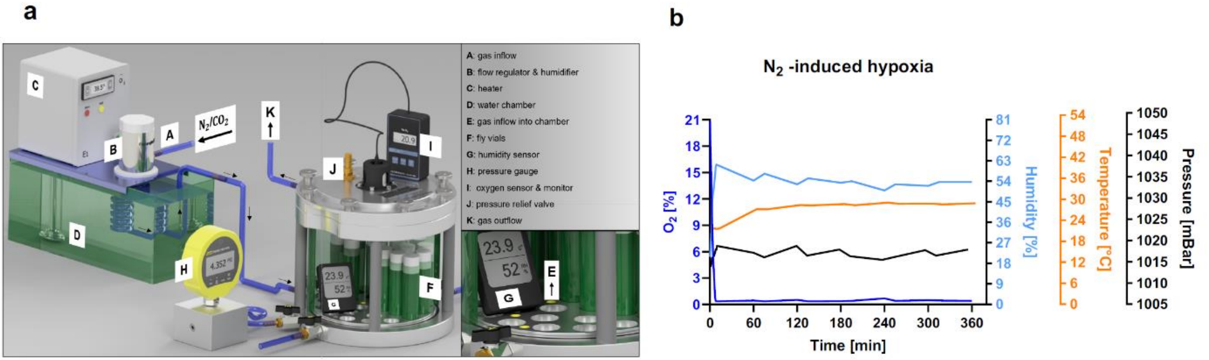

Supplemental Figure 1: Custom hypoxia chamber with the capability to monitor oxygen levels, temperature, humidity and pressure

(a) Model of a custom hypoxia chamber for *Drosophila melanogaster*. **(b)** Representative graph of environmental conditions (oxygen levels, temperature, humidity and pressure) monitored during hypoxia.

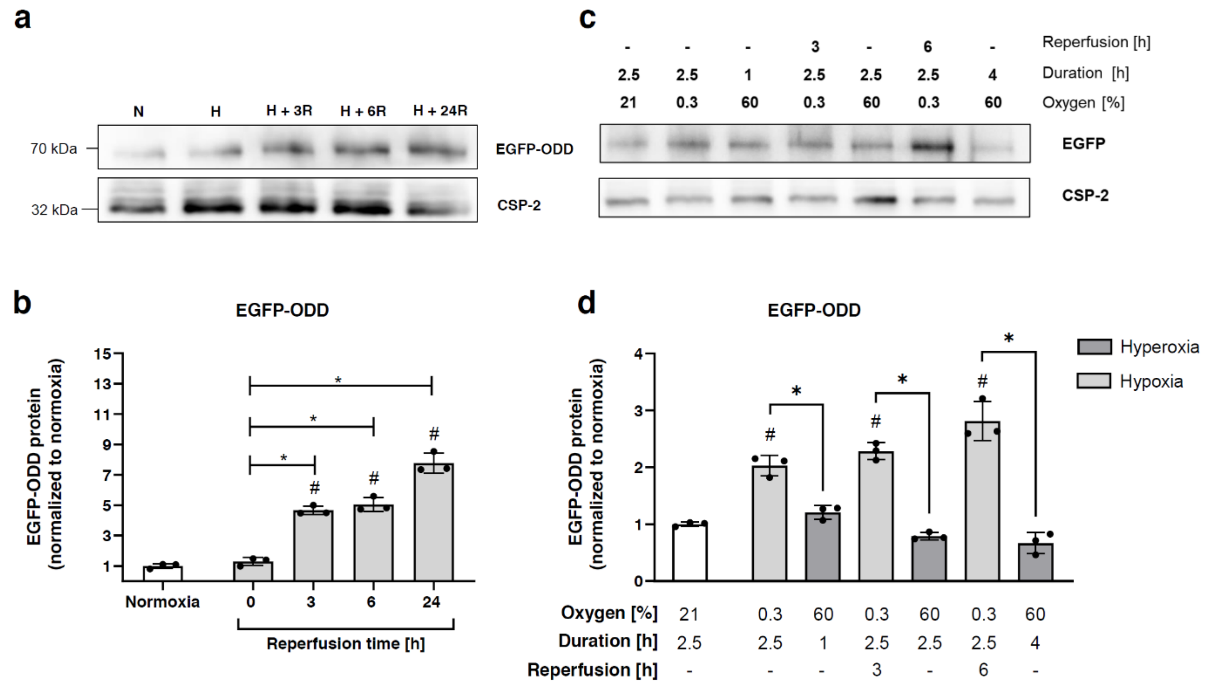

Supplemental figure 2: Hif1α/Sima protein levels after severe hypoxia (<0.3 % O_2) and hyperoxia (>60 % O_2).

(a) Representative Western blot showing the EGFP-ODD and CSP-2 protein levels after 2.5 h of severe hypoxia with various reperfusion periods. **(b)** Posthypoxic EGFP-tagged ODD protein levels as ratios to the CSP-2 levels after 0, 3, 6 and 24 h of reperfusion normalized to the levels in normoxia-exposed flies. **(c)** Representative Western blot showing the EGFP-ODD and CSP-2 protein levels after 2.5 h of severe hypoxia followed by reperfusion and various hyperoxia durations. **(d)** Posthypoxic and posthyperoxic EGFP-tagged ODD protein levels as ratios to CSP-2 levels normalized to the levels in normoxia-exposed flies. The data are presented as the means ± SEMs from 3 independent experiments. Kruskal-Wallis test followed by Dunn's multiple comparison test. *p<0.05, # indicates significance compared to normoxia. *p<0.05, * indicates intergroup comparison.

Author	Year	Journal	O_2 concentration	humidification of gas?	humidity measured?	temperature measured?	pressure measured?	reference [DOI]
Yiling Xia et al	2017	Neuroscience Bulletin	not stated	yes	yes	25 °C	no	10.1007/s12264-017-0173-7
Qui Fang Li et al	2020	Frontiers in Physiology	6%	yes	no	no	no	10.3389/fphys.2019.01610
Sassù et al	2019	PLOS ONE	0.3%	no	no	18 °C	no	10.1371/journal.pone.0226582
Yi-Wie Tsai et al	2019	PNAS	21, 10 & 1%	no	no	no	no	10.1073/pnas.1902537116
Pei-Yi Chen	2019	PLOS genetics	5%	no	no	no	no	10.1371/journal.pgen.1007980
Campbell et al	2019	AM J PHYSIOL-REG I	not stated	yes	no	25 °C	no	10.1152/ajpregu.00389.2018
Campbell et al	2019	G3	not stated	yes	no	no	no	10.1534/g3.119.400421
Xiao et al	2019	J EXP BIOL	not stated	no	no	no	no	10.1242/jeb.199521
Xiao et al	2019	Neuroscience Letters	not stated	no	no	no	no	10.1016/j.neulet.2019.04.053
Lee et al	2019	nature communications	5%	no	no	no	no	10.1038/s41467-019-09643-7
Cho et al	2018	nature communications	10%	no	no	25 °C	no	10.1038/s41467-018-04990-3
Cruz et al	2018	CHEM-BIOL INTERACT	3-4%	no	no	25 °C	no	10.1016/j.cbi.2018.06.033
Campbell et al	2018	J EXP BIOL	not stated	yes	no	25 °C	no	10.1242/jeb.177147
Teague et al	2017	Biology letters	12%	no	no	25 °C	no	10.1098/rsbl.2017.0309
Zarndt et al	2017	CIRC-CARDIOVASC GENE	4%	no	no	no	no	10.1161/CIRCGENETICS.117.001706
Evans et al	2017	COMP BIOCHEM PHYS A	not stated	no	no	no	no	10.1016/j.cbpa.2017.09.006
Wang et al	2017	PLOS one	0%	no	no	25 °C	no	10.1371/journal.pone.0185267
Batie et al	2017	cells	5 or 3%	no	no	no	no	10.3390/cells6010008
Mossmann et al	2017	MOL BIOL EVOL	6%	no	no	25 °C	no	10.1093/molbev/msw246
Xiao et al	2016	PLOS one	not stated	no	no	no	no	10.1371/journal.pone.0168361
Misra et all	2016	Biology Open	5%	no	no	22 °C	no	10.1242/bio.018226
Ezcurra et al	2016	PLOS one	11% & 5%	no	no	no	no	10.1371/journal.pgen.1006073
Bertolin et al	2016	nucleic acids research	5% & 8%	no	no	25 °C	no	10.1093/nar/gkw372
Xiao et al	2016	genetics	not stated	no	no	no	no	10.1534/genetics.115.185066
Jha et al	2015	MOL BIOL EVOL	8% - 5%	no	no	no	no	10.1093/molbev/msv248
Cruz et al	2015	neurochem Res	3-5%	no	no	25 °C	no	10.1007/s11064-015-1744-5
Dong et al	2015	Journal of cell biology	0.5%	no	no	25 °C	no	10.1083/jcb.201503067
Zarndt et al	2015	AM J PHYSIOL-REG I	4% & 1%	yes	no	room temperature	no	10.1152/ajpregu.00164.2015.
Callier et al	2015	J EXP BIOL	0.03 kPa	yes	yes	25 °C	no	10.1242/jeb.125849
Benasayag-Meszaros et al	2015	Scientific reports	0%	no	no	23 °C & 3 °C	no	10.1038/srep09204
Delalio et al	2015	Journal of insect biology	2%	no	no	no	no	10.1016/j.jinsphys.2015.02.009
Wong et al	2014	PLOS one	3.5 %	no	no	room temperature	no	10.1371/journal.pone.0115297
Gersten et al	2014	PLOS one	8% - 5% & 4%	no	no	no	no	10.1371/journal.pone.0103292

Bandarra et al	2014	Bioscience reports	3%	no	no	no	no	10.1042/BSR20140095
Udpa et al	2014	genome biology	5%	no	no	22 °C	no	10.1186/gb-2014-15-2-r36
Caraballo et al	2014	PLOS one	< 1%	no	no	no	no	10.1371/journal.pone.0084434
Sansone et al	2013	PLOS one	< 1%	no	no	no	no	10.1111/j.1601-183X.2011.00743.x
Van der Laan et al	2012	the international journal of developmental biology	1%	no	no	29 °C	no	10.1387/ijdb.103172al
Wen et al	2012	MOL CELL NEUROSCI	1%	no	no	no	no	10.1016/j.mcn.2012.11.008
Azad et al	2012	G3	5%	no	no	no	no	10.1534/g3.112.003681
Perkins et al	2012	PLOS one	4 kPa (4 %)	no	no	no	no	10.1371/journal.pone.0045344
Rodríguez et al	2012	J EXP BIOL	< 1 %	yes	no	yes	no	10.1242/jeb.074468
Azad et al	2011	FREE RADICAL BIO MED	1.5 %	no	no	no	no	10.1371/journal.pone.0005371
Heinrich et al	2011	J EXP BIOL	10 kPa, (10%)	yes	95%	no	no	10.1242/jeb.051904
Ali et al	2011	J Biol Chem	5%	no	no	no	no	10.1074/jbc.M111.219295
Schilman et al	2010	J EXP BIOL	not stated	no	no	20, 25 & 30 °C	no	10.1242/jeb.052357
Zhou et al	2010	PNAS	5%	no	no	22 °C	no	10.1073/pnas.1010643108
Mosqueira et al	2010	PLOS one	13% & 1%	no	no	no	no	10.1371/journal.pone.0013450
Vermehren-Schmaedick et al	2010	genetics	0, 5, 10, 15%	no	no	no	no	10.1534/genetics.110.118166
Vigne et al	2010	BMC physiology	5%	no	no	21 °C	no	10.1186/1472-6793-10-8
Whelan et al	2009	Elsevier Brain research	5 %, 2%, 1%	no	no	no	no	10.1016/j.brainres.2009.12.036
Voorhies et al	2009	J EXP BIOL	0, 0.1, 0.2, 1.0 k Pa	yes	no	no	no	10.1242/jeb.031179
Feala et al	2009	BMC systems biology	4%	yes	no	no	no	10.1186/1752-0509-3-91
Klok et al	2009	J. Insect Physiol.	10 - 0 kPa (10 - 0%)	yes	no	temperature sensor	no	10.1016/j.jinsphys.2009.08.004
Vigne et al	2009	PLOS one	0.1%	no	no	25 °C & 21 °C	no	10.1371/journal.pone.0005422
Azad et al	2009	PLOS one	1%	yes	30-50 %	22-24 °C	no	10.1371/journal.pone.0005371
Mortimer et al	2009	Developmental Biology	0.5%	no	no	no	no	10.1016/j.ydbio.2009.03.001
Klok et al	2009	PLOS one	10 kPa, (10%)	no	no	25 °C	no	10.1371/journal.pone.0003876
Coquin et al	2008	Mol. Syst. Biol.	0.5%	yes	no	25 °C	no	10.1038/msb.2008.71
Wick et al	2008	FREE RADICAL BIO MED	5%	no	no	25 °C	no	10.1016/j.freeradbiomed.2008.09.036
Zhou et al	2008	PLOS one	< 8%	yes	no	no	no	10.1371/journal.pgen.1000221
Centanin et al	2008	developmental cell	5%	no	no	no	no	10.1016/j.devcel.2008.01.020
Feala et al	2008	Ann. **N. Y.** Acad. Sci.	3%	no	no	26 °C	no	10.1196/annals.1420.019
Romero et al	2008	Mol Cell Biol	1%	no	no	25 °C	no	10.1128/MCB.01027-07

Vigne et al	2007	science direct	5%	no	no	25 °C	no	10.1016/j.exger.2007.09.012
Pandey et al	2007	Journal of cell science	2%	no	no	22-24 °C	no	10.1242/jcs.007690
Feala et al	2007	Mol. Syst. Biol.	0.5%	yes	no	25 °C	no	10.1038/msb4100139
Huang et al	2007	physiol. Genomics	4% & 0%	no	no	25 °C	no	10.1152/physiolgenomics.00166.2006
nilson et al	2006	J. Insect Physiol.	not stated	no	no	no	no	10.1016/j.jinsphys.2006.07.001
Liu et al	2006	physiol. Genomics	0.5% & 5%	no	no	22 °C & 25 °C	no	10.1152/physiolgenomics.00262.2005
Morin et al	2005	MOL CELL BIOCHEM	not stated	no	no	15 °C	no	10.1007/s11010-005-8236-x
Peck et al	2005	J EXP ZOOL	7.5%, 10%, 15%, 20%	yes	no	no	no	10.1002/jez.a.211.
Centanin et al	2005	EMBO reports	5%	no	no	25 °C	no	10.1038/sj.embor.7400528
Arguier et al	2006	biochem. J.	5%	no	no	no	no	10.1042/BJ20050675
Reiling et al	2004	Genes & Development	< 2.8%	no	no	25 °C	no	10.1101/gad.322704.
Douglas et al	2004	AM J PHYSIOL-REG I	0%, 5% & 2%	no	no	no	no	10.1152/ajpregu.00520.2004
Teodoro et al	2003	the EMBO Journal	not stated	no	no	no	no	10.1093/emboj/cdg070
Chen et al	2002	J Biol Chem	0%	no	no	no	no	10.1074/jbc.M109479200
Douglas et al	2001	AM J PHYSIOL-REG I	10 mmHg (1.3 kPa); 20 mmHg (2.6 kPA)	no	no	no	no	10.1152/ajpregu.2001.280.5.R1555
DiGregorio et al	2000	J Biol Chem	< 0.1%, 1% & 2%	yes	no	no	no	10.1074/jbc.M003911200
Ma et al	1999	molecular brain research	0% ; < 0.02%	yes	no	no	no	10.1016/s0169-328x(99)00224-7
Xia et al	1998	PHYSIOL BEHAV	not stated	yes		25 °C	no	10.1016/S0031-9384(98)00191-7
Ma et al	1998	molecular brain research	0% ; < 0.02%	yes	no	no	no	10.1016/s0169-328x(98)00265-4
Ma et al	1997	molecular brain research	< 0.02%	no	no	no	no	10.1016/s0169-328x(97)00074-0
KRISHNAN et al	1996	J. Insect Physiol.	0.03 %	no	no	no	no	10.1016/s0022-1910(96)00084-4
Foe et al	1985	Journal of cell biology	not stated	no	no	25 °C	no	10.1083/jcb.100.5.1623
Olney et al	1976	nature	not stated	no	no	no	no	10.1038/264661a0
Matheson et al	1973	THEOR APPL GENET	not stated	yes	no	25 °C	no	10.1007/BF00277786

Supplemental Table 1: Validation of hypoxia-induced Hif/Sima regulation in *D.m.*

A search for literature in May 2020 revealed 88 publications (excluding reviews) dealing with hypoxia, anoxia or ischemia in *Drosophila melanogaster.* The hypoxia protocols in these 88 publications with regard to oxygen concentration, temperature, humidity and pressure monitoring during hypoxia experiments are summarized.
